# Comparison of the Fecal Microbiota of Horses with Intestinal Disease and Their Healthy Counterparts

**DOI:** 10.3390/vetsci8060113

**Published:** 2021-06-17

**Authors:** Taemook Park, Heetae Cheong, Jungho Yoon, Ahram Kim, Youngmin Yun, Tatsuya Unno

**Affiliations:** 1 Equine Clinic, Jeju Stud Farm, Korea Racing Authority, Jeju 63346, Korea; taemook7@gmail.com (T.P.); junghoy11@gmail.com (J.Y.); aidia0207@naver.com (A.K.); 2 College of Veterinary Medicine and Veterinary Medical Research Institute, Jeju National University, Jeju 63243, Korea; 3 College of Veterinary Medicine and Institute of Veterinary Science, Kangwon National University, Chuncheon 24341, Korea; htcheong@kangwon.ac.kr; 4 Faculty of Biotechnology, School of Life Sciences, SARI, Jeju 63243, Korea; 5 Subtropical/Tropical Organism Gene Bank, Jeju National University, Jeju 63243, Korea

**Keywords:** fecal microbiota, colic, NGS, thoroughbred, methanogen

## Abstract

(1) Background: The intestinal microbiota plays an essential role in maintaining the host’s health. Dysbiosis of the equine hindgut microbiota can alter the fermentation patterns and cause metabolic disorders. (2) Methods: This study compared the fecal microbiota composition of horses with intestinal disease and their healthy counterparts living in Korea using 16S rRNA sequencing from fecal samples. A total of 52 fecal samples were collected and divided into three groups: horses with large intestinal disease (*n* = 20), horses with small intestinal disease (*n* = 8), and healthy horses (*n* = 24). (3) Results: Horses with intestinal diseases had fewer species and a less diverse bacterial population than healthy horses. Lactic acid bacteria, Lachnospiraceae, and Lactobacillaceae were overgrown in horses with large intestinal colic. The Firmicutes to Bacteroidetes ratio (F/B), which is a relevant marker of gut dysbiosis, was 1.94, 2.37, and 1.74 for horses with large intestinal colic, small intestinal colic, and healthy horses, respectively. (4) Conclusions: The overgrowth of two lactic acid bacteria families, Lachnospiraceae and Lactobacillaceae, led to a decrease in hindgut pH that interfered with normal fermentation, which might cause large intestinal colic. The overgrowth of *Streptococcus* also led to a decrease in pH in the hindgut, which suppressed the proliferation of the methanogen and reduced methanogenesis in horses with small intestinal colic.

## 1. Introduction

Horses are nonruminant herbivores whose digestive system has evolved to utilize the fibers in the roughages in their hindgut [1,2,3,4]. The large intestine of horses is an anaerobic fermentation chamber filled with fibrolytic bacteria. Therefore, the large intestinal microbiota of horses plays an essential role in the utilization of plant fibers by producing volatile fatty acids (VFAs), such as acetate, propionate, and butyrate, which are absorbed through the cecal and colonic epithelium and distributed for use throughout the body [5]. In addition to metabolic benefits, the intestinal microbiota provides the host with other advantages, including protection against pathogen overgrowth, stimulation of the immune response in the gut, and enhanced intestinal barrier function by regulating gene expression in the host intestinal epithelial tissue [6,7,8,9,10,11].

The relationship between the gut microbiota and clinical conditions, such as inflammatory bowel disease, colorectal cancer, or diabetes, have been examined in large-scale studies involving humans [12,13,14,15,16]. Studies performed on different animal species have shown that the gut microbial dynamics can be influenced by various factors, including the environment, diet, gestational age, hospitalization, antibiotics, delivery mode, stress, and feeding method [7,8,9,12,17,18]. Similarly, differences in the fecal bacterial communities between horses with gastrointestinal diseases and their healthy counterparts have been reported [6,19,20,21,22]. Furthermore, an acute change in the colonic microbiota was observed in horses that underwent an exploratory laparotomy to treat equine colic [23]. A disturbance in the equine hindgut microbiota can alter the fermentation patterns and ultimately lead to metabolic disorders [24].

Carbohydrate fermentation is the main source of lactic acid production in the equine hindgut. In healthy horses, the luminal lactate is converted to VFAs by commensal bacteria. Therefore, very little lactate is present in their hindgut [25]. Rapid dietary changes, such as grain overload, have long been recognized to disrupt normal fermentation in the horse hindgut. Consequently, excessive carbohydrate fermentation leads to lactate accumulation in the hindgut, which can induce subclinical acidosis. Low pH in the gut lumen alters the hindgut microbiota, which causes the release of endotoxin from the death of acid-sensitive Gram-negative bacteria and compromises the intestinal barrier function. Such changes linked to development of potentially life-threatening complications include colitis [6,22,26], laminitis [11,20,27,28], and systemic inflammatory response syndrome [29].

Alteration of the equine hindgut microbiota in clinical conditions has been reported in many studies [2,6,11,19,20,21]. Similarly, discrepancies in the hindgut microbiota in horses with intestinal disease and healthy horses have been reported [2,6,20,21,22]. On the other hand, the associations of equine hindgut microbiota with health and disease have not been entirely understood [6,20,21,22,26,30]. The present study was conducted to determine the differences between the horse fecal microbiota of healthy and diseased horses. In this study, we differentiated the diseased horses in large and small intestinal colic diseased animals. Our results further contribute to our understanding of how horse fecal microbiota is associated with different intestinal colic disorders.

## 2. Materials and Methods

### 2.1. Horse Descriptions and Fecal Sampling

All animal protocols were approved by the Institutional Animal Care and Use Committee of Korea Racing Authority (KRA IACUC-2009-AEC-2007). A total of 28 adult horses admitted to the Jeju Stud Farm Equine Clinic of Korea Racing Authority to evaluate gastrointestinal diseases were included in this study.

The 28 horses showing signs of colic were divided further into two study groups: horses with large intestinal colic (LC, *n* = 20) and horses with small intestinal colic (SC, *n* = 8). A total of 24 clinically healthy adult horses (HH, *n* = 24) from seven independent farms in Jeju island, Korea, were also included in the study (6.2 ± 3.1 years, 8 male, 14 female, 2 gelded). The horses in the control group did not receive any antimicrobials or anti-inflammatory drugs. They had no history of gastrointestinal diseases for the two months prior to the study. All horses included in this study were thoroughbreds.

The fecal samples were collected from horses with gastrointestinal diseases within two hours after admission to the clinic. The fecal samples were collected directly from the rectum to minimize environmental contamination using clean rectal gloves and sterile lubrication (Kruuse, Langeskov, Denmark) as described previously [31]. Each sample was placed in a sealed collection bag and stored at −80 °C until DNA extraction. Fresh fecal samples were obtained from the healthy control horses in a similar manner.

### 2.2. Microbial Community Analysis

The fecal DNA was extracted using a PowerFecal DNA extraction kit (Qiagen, Hilden, Germany). The V3 and V4 regions of the partial 16S rRNA gene were amplified by a polymerase chain reaction (PCR) using the 341F (5′-TCGTCGGCAGCGTCAGATGTGTATAAGAGACAGCCTACGGGNGGCWGCAG-3′) and 806R (5′-GTCTCGTGGGCTCGGAGATGTGTATAAGAGACAGGACTACHVGGGTATCTAATCC-3′) primer sets. Two-step PCR was performed to construct the MiSeq library. Sequencing was performed at Macrogen Inc. (Seoul, Korea) according to the manufacturer’s instruction. The sequence data were processed using MOTHUR according to the standard operational protocol as previously described online (https://mothur.org/wiki/miseq_sop/) (accessed on 14 April 2021) with a minor modification of singleton removal after the pre.cluster subroutine. Silva.nr_v132 was used for alignment, and RDP version 11.5 was used for the taxonomic classification. The operational taxonomic units (OTUs) were assigned using the opti.clust algorithm with a sequence distance at 0.03. PICRUSt2 was used to predict the metabolic activities based on 16S rRNA gene sequences. The MetaCyc database [32] was used to define the differentially abundant metabolic pathways indicated by PICRUSt2.

### 2.3. Statistics

MOTHUR was used to calculate the ecological indices (Chao I and Shannon) for species richness and evenness. Nonmetric multidimensional scaling (NMDS) was performed and plotted with ellipses at the 95% confidence level using the vegan R package. MOTHUR was used to analyze the molecular variances (AMOVA) to determine the significant differences in fecal microbiota in the study. Differential abundance analysis was performed using the liner discriminant analysis effect size (LEfSe) and ALDEx2 for the OTUs and predicted metabolic activities, respectively. A Wilcoxon rank-sum test was applied to compare the ecological indices. The differences were considered significant at *p* < 0.05.

## 3. Results

### 3.1. α-Diversity Analysis

**Figure 1:**
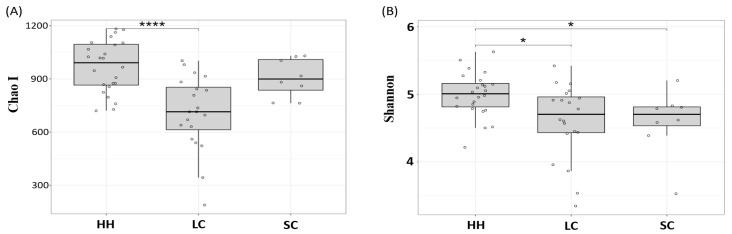
Comparison of the horse fecal microbiota ecological indices for species richness using Chao I (**A**) and species evenness using Shannon (**B**). Significant mean difference evaluated by Wilcoxon test are indicated with * and **** for *p* < 0.05 and *p* < 0.0001, respectively. HH, healthy horses; LC, horses with large intestinal colic; SC, horses with small intestinal colic.

The differences in the alpha-diversities between horses with intestinal disease and healthy horses were analyzed using the Chao I and Shannon indices for species richness and evenness estimation, respectively. All samples showed a Good’s coverage greater than 98%, suggesting that sequence depth was sufficient to cover most of the species in the samples (Figure S1). The species richness of the horses with large intestinal colic was lower than that of healthy horses (*p* < 0.0001). In contrast, there was no difference between healthy horses and horses with small intestinal colic (Figure 1A). On the other hand, the species evenness was lower in both colic groups compared to the healthy horses (Figure 1B) (*p* < 0.05). These results suggest that the intestinal disease status affects the alpha-diversity of the fecal microbiota.

### 3.2. β-Diversity and Taxonomic Composition Analysis

**Figure 2:**
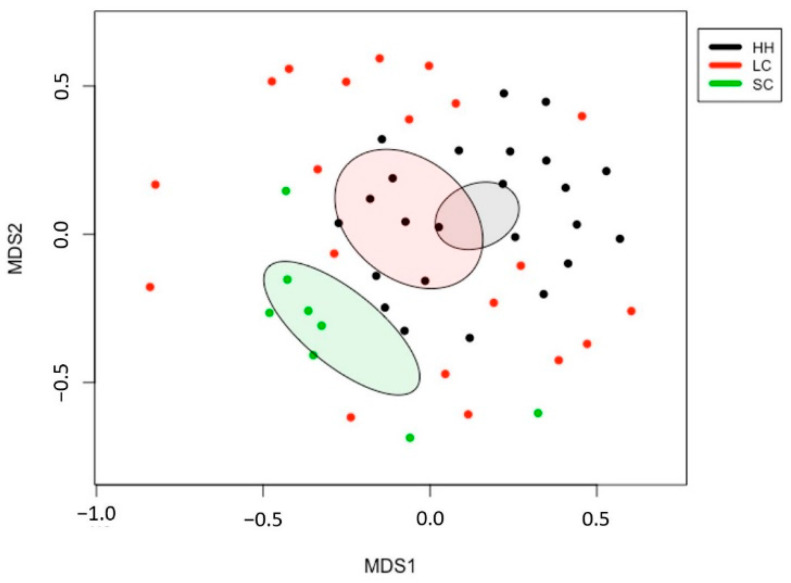
Nonmetric multidimensional scaling (NMDS) analysis for a beta-diversity comparison of the horse fecal microbiota. HH, healthy horses; LC, horses with large intestinal colic; SC, horses with small intestinal colic.

The fecal microbiota of horses with small intestinal colic was more distant from healthy horses or horses with large intestinal colic (Figure 2). AMOVA revealed significant differences in the intestinal microbiota (*p* < 0.01). A comparison of the fecal microbial communities at the phylum level in previous studies showed that healthy horses possessed a consistent portion of Firmicutes and Bacteroidetes [33,34,35,36,37], whereas a high abundance of Firmicutes was observed among horses with small intestinal colic. 

**Figure 3:**
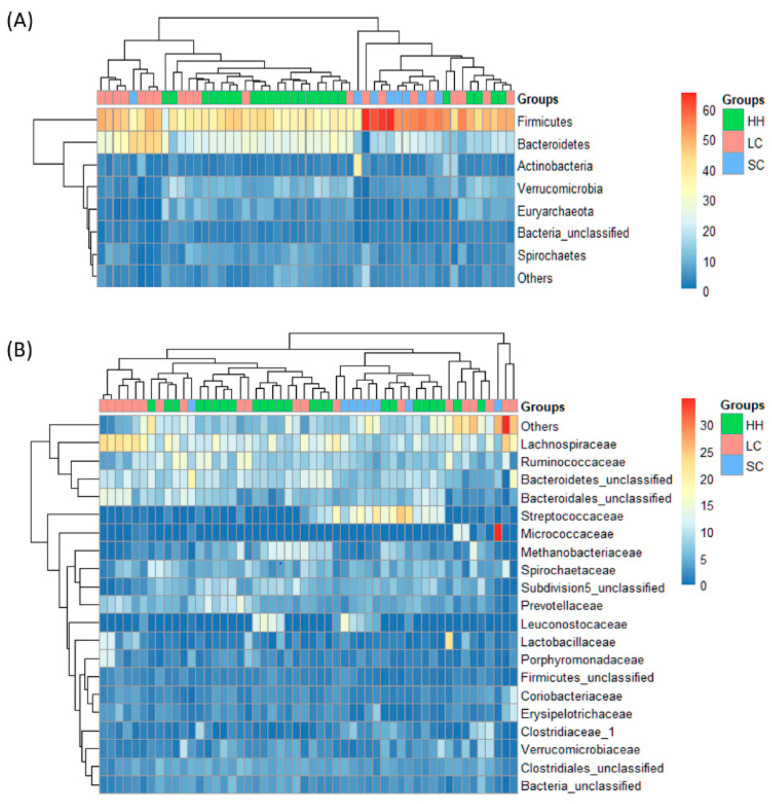
Comparison of the horse fecal microbiota bacterial composition at the phylum (**A**) and family (**B**) level. HH, healthy horses; LC, horses with large intestinal colic; SC, horses with small intestinal colic.

Horses with large intestinal colic appeared to have a higher abundance of Bacteroidetes and a lower abundance of Verrucomicrobia than healthy horses (Figure 3A). At the family level (Figure 3B), a higher abundance of Lachnospiraceae and Streptococcaceae was observed in horses with large intestinal colic, and horses with small intestinal colic had a significantly lower Subdivision5_unclassified family belonging to the phylum Verrucomicrobia compared to healthy horses (*p* < 0.05). Lactobacillaceae and Coriobacteriaceae were significantly more abundant in horses with large intestinal colic than healthy horses (*p* < 0.05). On the other hand, Methanobacteriaceae was significantly lower in horses with small intestinal colic than healthy horses (*p* < 0.05).

### 3.3. Differentially Abundant Genera

**Figure 4:**
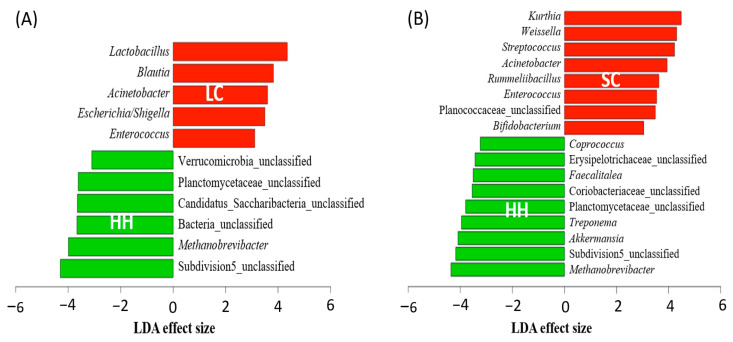
Differentially abundant genera in horse fecal microbiota among LC (**A**) and SC (**B**) compared to HH. HH, healthy horses; LC, horses with large intestinal colic; SC, horses with small intestinal colic.

The differentially abundant genera in each group were identified by LEfSe (Figure 4). In horses with intestinal disease groups, the density of *Enterococcus* and *Acinetobacter* were increased significantly, whereas the presence of *Methanobrevibacter* was reduced significantly (*p* < 0.05). Interestingly, the well-known probiotics *Lactobacillus* and *Bifidobacterium*, which are commonly known probiotics in horses, were increased in horses with large intestinal colic and small intestinal colic, respectively. Some *Blautia*, *Enterococcus*, and *Streptococcus* species were more abundant in horses with intestinal disease, even though these are known as probiotics for humans [38,39,40]. In horses with small intestinal colic, genera *Kurthia*, *Weissella*, and *Rummeliibacillus* were abundant, but their roles in the gut are yet to be discovered. Most of the fecal microbiota genera that were reduced in horses with intestinal disease were unclassified genera except for *Methanobrevibacter* in both horses with large intestinal colic and small intestinal colic and *Coprococcus*, *Faecalitalea*, *Treponema*, and *Akkermansia* in horses with small intestinal colic. *Treponema* is a known human pathogen, whereas *Akkermansia* and *Coprococcus* are beneficial to humans. *Faecalitalea* is not a well-known bacteria.

### 3.4. Comparison of the Metabolic Activities between Horses with Intestinal Disease and Healthy Horses

Table 1 and Table 2 list the significantly enriched and depleted metabolic activities in horses with small intestinal colic compared to healthy horses, respectively. The enriched pathways were involved in two functions, enterobactin biosynthesis and the TCA cycle. On the other hand, the depleted pathways were also involved in two functions, methanogen lipid membrane biosynthesis and methanogenesis. Because both functions are related to methanogenic bacteria, likely *Methanobrevibacter*, a loss of *Methanobrevibacter* may be associated with small intestinal colic. Nevertheless, the intestinal metabolic activities were similar in healthy horses and those with large intestinal colic.

## 4. Discussion

As demonstrated in other studies [19,21,29], this study confirms that the bacterial community compositions of horses with intestinal diseases are considerably different from that of their clinically healthy counterparts. In particular, horses with large intestinal colic had lower species evenness and richness than the healthy horses, with some bacterial species no longer detectable and the generation of greater evenness.

In this study, a clear difference in the bacterial compositions was observed between horses with intestinal disease and healthy horses at the phylum level. The Firmicutes to Bacteroidetes (F/B) ratio, which has been reported to indicate gut dysbiosis in humans [41], was increased in horses with an intestinal condition compared to healthy controls, which is consistent with previous reports [21,42]. In addition, the average F/B ratios were 1.94, 2.37, and 1.74 for horses with large intestinal disease, small intestinal disease, and healthy controls, respectively. In contrast to previous reports, increased Bacteroidetes in horses admitted for colic was not observed in the current research [19,42]. This suggests that the F/B ratio alone is not enough to evaluate the intestinal disease status of horses.

At the family level, horses with large intestinal illnesses had larger numbers of two lactic acid bacteria, Lachnospiraceae and Lactobacillaceae. This observation may support previous findings that excessive lactate production and decreases in the hindgut luminal pH are associated with an increased relative abundance of *Streptococcus* and lactic acid bacteria in horses with colic. Furthermore, a decrease in luminal pH negatively affects fiber digestion and volatile fatty acid production [11,43,44], which might have decreased Methanobacteriaceae. This is likely because methanogens are quite sensitive to the acidic environment, as reported previously [45].

At the genus level, overgrowth of *Lactobacillus* or *Streptococcus* was observed in horses with colic with large or small intestinal origin, respectively. Similar to the current findings, an increased abundance of lactic acid bacteria was reported to be a major cause of intestinal dysbiosis and colic [11,43,46]. Moreover, a decrease in pH may decrease the abundance of methanogens in intestinal disease horses [47]. In the current study, both *Escherichia* and *Streptococcus* were increased in horses with intestinal diseases, similar to previously reported findings [11,26,44]. In detail, *Escherichia* was increased markedly in horses with large intestinal colic, while an increased number of *Streptococcus* was noted in horses with small intestinal disease [11,44].

Although the beneficial effects of *Lactobacillus* and *Bifidobacterium* are well documented in humans [48], both genera were more abundant in horses with colic in the current study. Such a discrepancy may suggest that the role of specific microbes can vary in different animal species. Further investigation on equine fecal microbiota and their functional role in health and disease will be needed to understand the benefits and dynamics of probiotics of horses.

Moreover, the abundance of *Methanobrevibacter* could be used to monitor the health status of horses because the results showed that *Methanobrevibacter* decreased significantly in horses with large and small intestinal colic compared to healthy horses. Methanogenic archaea are often abundant in healthy equine colon [49], which metabolizes H_2_ and CO_2_ to produce methane and likely supports the degradation of cellulolytic bacteria in the lower gut [50,51]. These results indicate that the abundance of *Methanobrevibacter* is associated with a healthy horse status. Steinberg and Regan reported that the quantification of methyl coenzyme M reductase α-subunit (mcrA) genes by real-time PCR successfully quantified different phylogeny of methanogens [52]. Further investigations with such qPCR-based quantification of methanogenic bacteria and diagnostics of horse physiological conditions, such as colic, enteritis, and other metabolic diseases, could verify if the abundance of methanogens can be used to indicate horse intestinal health.

While several genera were differentially abundant between horses with large intestinal colic and healthy horses, no significant difference was observed in intestinal metabolic activities predicted by PICRUSt2. This might be because nonhuman samples are less accurate in predicting metabolic activities through PICRUSt algorithms. On the other hand, metabolic activity prediction also indicate that decreased methanogenic activities and increased activities of enterobactin biosynthesis are associated with the disease status of horses.

The limitations of this study include the small number of horses recruited in colic groups. A follow-up study using a larger number of horses with different types of intestinal disease will be needed to better understand the differential distribution of microbiota richness in different pathogenesis of colic.

## 5. Conclusions

The bacterial community composition in horses with intestinal disease was substantially different from that of healthy horses. Horses with intestinal disease had fewer species and a less diverse bacterial population than healthy horses. The overgrowth of lactic acid-producing bacteria, such as Lachnospiraceae, Lactobacillaceae, and even probiotics for humans, can decrease the hindgut pH, which subsequently interferes with fermentation and produces excessive gas in the hindgut, eventually causing large intestinal colic. The abundance of methanogen, however, might be negatively associated with horse intestinal health.

## Figures and Tables

**Table 1:** Enriched metabolic pathways in the SC compared to the HH.

Pathways (MetaCyc)	Enriched Pathways in SC	ALDEx Diff.	Metabolism
ALL-CHORISMATE-PWY	Superpathway of chorismate metabolism	8.16	Enterobactin biosynthesis
ENTBACSYN-PWY	Enterobactin biosynthesis	8.02	
PWY0-321	Phenylacetate degradation I (aerobic)	5.05	TCA cycle
PWY-5178	Toluene degradation IV (aerobic) (via catechol)	5.01	
PWY-6185	4-methylcatechol degradation (ortho cleavage)	4.77	
PWY0-1277	3-phenylpropanoate and 3-(3-hydroxyphenyl) propanoate degradation	4.60	
PWY-5417	Catechol degradation III (ortho-cleavage pathway)	4.53	
PWY-6182	Superpathway of salicylate degradation	4.38	
PWY-5431	Aromatic compounds degradation via & beta;-ketoadipate	4.21	
TCA-GLYOX-BYPASS	Superpathway of glyoxylate bypass and TCA	4.11	

HH, healthy horses; SC, horses with small intestinal colic.

**Table 2:** Depleted metabolic pathways in the SC compared to the HH.

Pathways (MetaCyc)	Depleted Pathways in SC	ALDEx Diff.	Metabolism
PWY-6141	Archaetidylserine and archaetidylethanolamine biosynthesis	−2.52	Methanogen lipid membrane biosynthesis
PWY-7286	7-(3-amino-3-carboxypropyl)-wyosine biosynthesis	−2.39	
PWY-6167	Flavin biosynthesis II (archaea)	−2.35	
PWY-6350	Archaetidylinositol biosynthesis	−2.32	
PWY-6349	CDP-archaeol biosynthesis	−2.31	
P261-PWY	Coenzyme M biosynthesis I	−2.49	Methanogenesis
METHANOGENESIS-PWY	Methanogenesis from H_2_ and CO_2_	−2.40	
PWY-6148	Tetrahydromethanopterin biosynthesis	−2.29	
PWY-5198	Factor 420 biosynthesis	−2.27	
TCA-GLYOX-BYPASS	Coenzyme B biosynthesis	−2.21	

HH, healthy horses; SC, horses with small intestinal colic.

## Data Availability

Publicly available datasets were analyzed in this study. These data are available in the NCBI SRA database (accession number; PRJNA728810).

## Supplementary Materials

The following are available online at https://www.mdpi.com/article/10.3390/vetsci8060113/s1, Figure S1: Good’s coverage obtained for each horse fecal sample in this study.
