# Supplementary material for: Comparison of the Fecal Microbiota of Horses with Intestinal Disease and Their Healthy Counterparts
Source: Vet Sci. 2021 Jun 17;8(6):113. doi: 10.3390/vetsci8060113 (PMC8234941; doi:10.3390/vetsci8060113)
Supplement: Supplementary file 1 [file vetsci-08-00113-s001.zip › vetsci-1210033-supplementary.pdf]

cancers

MDPI

Supplementary Materials

Comparison of the Fecal Microbiota of Horses with Intestinal Disease and Their Healthy Counterparts

Taemook Park, Heetae Cheong, Jungho Yoon, Ahram Kim, Youngmin Yun and Tatsuya Unno

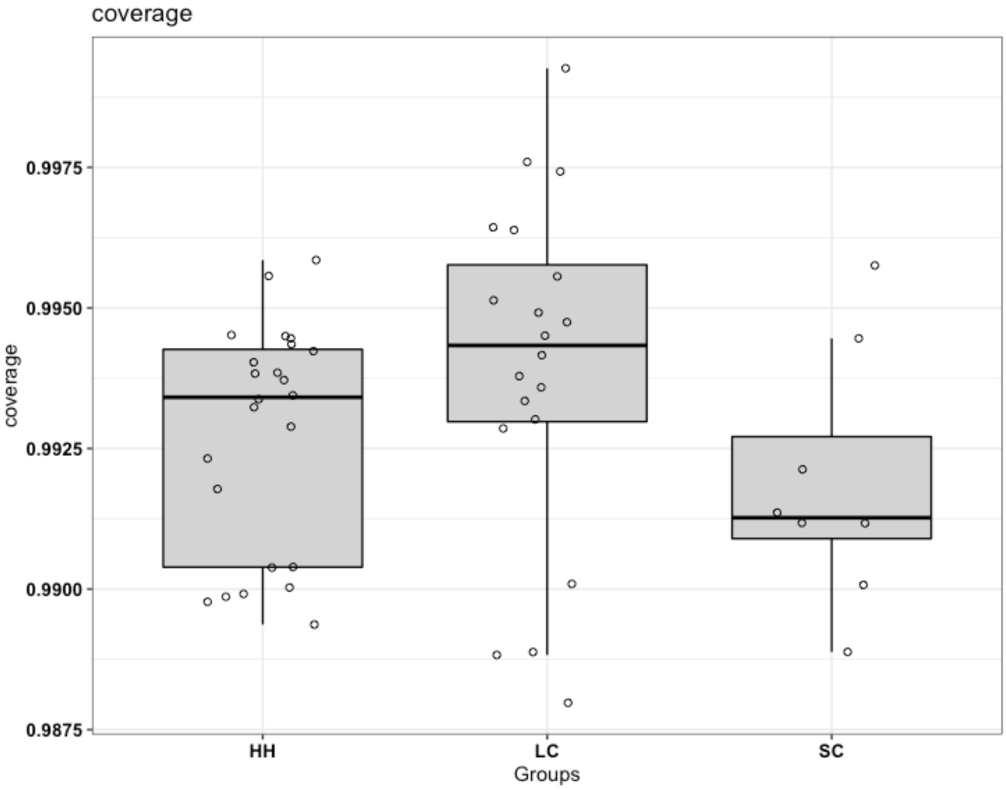

Figure S1. Good's coverage obtained for each horse fecal sample in this study.
